# Tailored Online Physical Activity Coaching for Middle-Aged and Older Adults With Cognitive and Mental Health Concerns: Single-Arm Pre-Post Intervention Study

**DOI:** 10.2196/80040

**Published:** 2026-03-06

**Authors:** Kathryn A Ellis, Rhoda Lai, Eleanor Curran, Jennifer Southam, Rebecca Moorhead, Kay L Cox, Serafino G Mancuso, Alissa Westphal, Terence W H Chong, Thomas Rego, Victoria J Palmer, Kaarin J Anstey, Nicola T Lautenschlager

**Affiliations:** 1 Department of Psychiatry, The University of Melbourne, Level 3, Alan Gilbert Building, 161 Barry Street, Parkville, Victoria, 3053, Australia, 61 383441879, 61 383441879; 2 Melbourne School of Psychological Sciences, The University of Melbourne, Parkville, Victoria, Australia; 3 St Vincent's Hospital Melbourne, Kew, Victoria, Australia; 4 Older Adult Mental Health Program, The Royal Melbourne Hospital, Melbourne, Victoria, Australia; 5 Murdoch Children's Research Institute, Parkville, Victoria, Australia; 6 Medical School, The University of Western Australia, Perth, Western Australia, Australia; 7 The ALIVE National Centre for Mental Health Research Translation, The University of Melbourne, Parkville, Victoria, Australia; 8 The Department of General Practice and Primary Care, The University of Melbourne, Parkville, Victoria, Australia; 9 School of Psychology, University of New South Wales, Sydney, New South Wales, Australia; 10 Neuroscience Research Australia, Randwick, New South Wales, Australia

**Keywords:** physical activity, exercise, older adults, aging, depression, anxiety, cognitive impairment, subjective cognitive decline, online intervention, coaching

## Abstract

**Background:**

Low levels of physical activity (PA) increase dementia risk, and for middle-aged and older adults with co-occurring cognitive concerns and mental health symptoms, dementia risk increases further. Despite clinical trials showing lower adherence to PA interventions in high-risk groups, there is a sparsity of interventions tailored to support unique behavior change needs. In EXCEL (Exercise for Cognitive Health) phase 1, we developed a model to understand the needs of this population and identified tailoring requirements to enhance engagement. Here we report the findings of a pilot online intervention designed to support middle-aged and older adults with subjective cognitive decline or mild cognitive impairment and mild to moderate symptoms of depression or anxiety to meet PA guidelines.

**Objective:**

We aimed to measure (1) efficacy of the intervention in promoting adoption of PA in line with national guidelines, (2) acceptability, feasibility, and safety of the intervention, (3) changes to dementia risk, PA levels, mental health symptoms, and stages of change, and (4) changes in potential cognitive mechanisms of change.

**Methods:**

A pilot individual 12-week online home-based PA intervention. Participants aged 45-80 years, experiencing both cognitive and mild-to-moderate anxiety and depression symptoms, were prescribed individually tailored PA programs combining aerobic and strength PA, plus balance training as indicated, with fortnightly online coaching.

**Results:**

A total of 55 participants were enrolled (46 females/9 males; mean age 62.2, SD 7.6 y). The intervention was effective; at baseline, only 3 of 55 (6%) participants met all applicable PA guidelines, compared to 24 of 55 (44%) participants post intervention (*P*<.001). Participants were at least 8 times more likely to meet the age-appropriate guidelines for aerobic, strength, and balance activities after the intervention. Retention rates were high (95% completion rate), and feedback indicated 98% found the program useful. Safety was successfully monitored by a clinical panel via email, using a system of alerts. Dementia risk was reduced (*d*=−0.32, *P*=.008), and reductions in depression, anxiety, and stress scores were large and clinically meaningful (*d*=−1.31, *d*=−0.89, and *d*=−1.18, respectively; all *P*<.001). Over half of the cohort (27 participants) transitioned to a higher stage of change at postintervention. Improvements in action planning (*d*=0.66, *P*<.001) and positive outcome expectancies (*d*=0.33, *P*=.01) indicate potential cognitive mechanisms of change.

**Conclusions:**

Our findings showed that this tailored, home-based online intervention successfully supports an at-risk cohort of aging adults to adopt PA, in line with national guidelines. The intervention was acceptable, feasible, and safe and led to significant reductions in dementia risk and mental health symptoms and was associated with progression through stage of change. Reflecting the Capability, Opportunity, and Motivation Behavior model underlying the intervention design, improvements in action planning and outcome expectancy appeared to play a role in bridging the PA intention-behavior gap.

## Introduction

Low physical activity (PA) levels are an established modifiable risk factor for dementia [1-3]. Depression and anxiety are also linked to dementia risk [4,5] and are associated with low levels of PA and other dementia risk factors [6]. The presence of cognitive concerns further adds to dementia risk, beyond the contribution of mood symptoms [7-9]. Accordingly, aging individuals who are concerned about their cognition and experience depressive or anxiety symptoms are considered an at-risk group for dementia [1,3,10]. The World Health Organization has emphasized the critical importance of directing dementia risk reduction (DRR) implementation research toward such at-risk populations, recognizing their heightened vulnerability and the urgent need for tailored and targeted strategies [11].

In 2018, our group developed PA guidelines for older adults living with mild cognitive impairment (MCI) or subjective cognitive impairment [12], complementing existing adults’ and older adults’ PA guidelines [13,14]. These guidelines recommend 150 minutes of moderate aerobic PA plus 2 strength sessions (of approximately 30 min each) per week, along with balance exercises as often as possible for older adults [12,13]. However, research shows that consumer awareness of PA guidelines is very low; none of the middle-aged to older adults interviewed by our team were aware of the guidelines [15], consistent with similarly low awareness internationally [16,17]. Implementation research is now vital to ensure individuals in middle to later life with mental and cognitive health symptoms can benefit from these guidelines, and from DRR interventions more broadly.

Symptoms of depression and anxiety, however, are associated with lower adherence and poorer outcomes in both PA and DRR intervention trials, as well as being a barrier to participation [6,18-20]. Tailored interventions for this population are essential to reduce dementia risk [11,21]. In a prior study [14], our team conducted a comprehensive analysis integrating data from semistructured interviews with 21 participants, a review of existing literature, and the Capability, Opportunity, and Motivation Behavior (COM-B) model to identify three behavior-change areas critical for this high-risk group: (1) emotional regulation, (2) overcoming barriers to turning intention into action, and (3) building confidence in one’s abilities [14]. These findings informed a model outlining strategies for tailoring PA interventions to the emotional and cognitive needs of this cohort. We additionally identified engagement preferences with information and communications technology [15,22], which was seen as essential to overcome access barriers [23], and technology was frequently cited as a useful tool by our target group for the accountability and connection they sought from PA interventions [15]. Indeed, older people appear to be well placed to benefit from PA interventions, given that we have seen relatively high and rising information and communications technology use in this age group [24]. We used this model [14] as the foundation for developing the EXCEL (Exercise for Cognitive Health) intervention. The PA programs themselves were based on our established work, which has successfully prescribed PA plans to older adults [25]. The EXCEL intervention was finalized at the beginning of a period of COVID-19–related lockdown in Victoria, Australia. As social isolation is linked to heightened experiences of symptoms of depression and anxiety [26,27] and a higher prevalence of these conditions [28], interventions for individuals with poorer mental health became more crucial than ever, and PA engagement and delivery were essential.

In this paper, we present the findings of EXCEL, a tailored 12-week home-based PA-delivered intervention designed to help middle-aged and older adults living with mild to moderate symptoms of depression, anxiety, or stress and cognitive concerns in adopting PA guidelines for DRR. Our primary hypothesis was that EXCEL would be associated with improved adherence to PA guidelines. We further hypothesized that the program would be acceptable, feasible, and safe, and associated with reductions in dementia risk and mood symptoms, and promote progression through stages of change. We further aimed to explore whether the intervention impacted potential cognitive mechanisms of behavior change.

## Methods

### Study Design

A single-arm pilot study of a 12-week online intervention combining PA prescription and individualized coaching. The current study measured the efficacy of the intervention for helping participants to meet age-appropriate PA guidelines [12,14], with secondary outcomes exploring acceptability, feasibility, and safety, and whether the intervention modified dementia risk, overall PA levels, mental health symptoms, and stages of change. We examined pre-post changes in measures of cognitive mechanisms of behavior change.

### Ethical Considerations

This study received ethical approval from the University of Melbourne Human Research Ethics Committee (2021-20479-16470-3). All published data is deidentified. Participants provided written informed consent to take part in this study before screening. Participants kept all programs and equipment provided during this study but did not receive other compensation for participating.

### Participants

Participants were eligible for inclusion if they were aged between 45 and 80 years; English-speaking; living in the community; had access to a phone and/or internet; reported concerns about their cognition (with or without objective cognitive decline) but not dementia; and experienced mild to moderate symptoms of depression, anxiety, or stress (measured using the short-form Depression Anxiety Stress Scales [DASS-21] [29]). Concerns about cognition were reported by a “yes” response to either of the questions: “Do you think your memory or thinking has changed?” or “Do you think your memory or thinking is worse than for other people your age?” Exclusion criteria were lack of capacity to provide informed consent; inability to read or write in English; diagnosis of dementia or a score indicative of dementia (27 or lower) on the Telephone Interview for Cognitive Status [30]; scores in the severe category or higher on either the depression, anxiety, or stress dimensions of the DASS-21 [29]; acute or past history of significant mental illness; current suicidality or history of suicidal attempts within the past 2 years; significant uncorrected sensory impairment; severe mobility impairment (either an inability to walk unaided or a score of 12 or more, indicative of medium to high risk, on the Falls Risk Assessment Tool [31]); any other significant medical condition that would prevent safely undertaking moderate-intensity PA (gauged using the Adult Pre-Exercise Screening System [32] and the Physical Activity Readiness Questionnaire [33]); or a BMI of less than 18.5 or 38 or higher (indicating that they were underweight or very obese). Where applicable, ethnically adjusted BMI indicators were used [34]. Participants were recruited from various sources across Australia, including state-wide seniors’ newsletters, university media, general practitioners, online self-registration services (StepUp platform) [35], and a database of volunteers who previously consented to be approached for research. Our target sample size was informed by a 1-sample proportion test [36] based on the AIBL Active Study [25]. To detect a 15% increase in participants meeting moderate-intensity PA guidelines with α=.05 and an expected attrition rate of 30% [37], we calculated a required sample size of 70, which would yield power of 0.80 to detect this effect.

### Intervention

EXCEL was a 12-week home-based PA intervention comprising the following resource and support components.

### PA Equipment, Guide, and Programs

**Figure 1.**
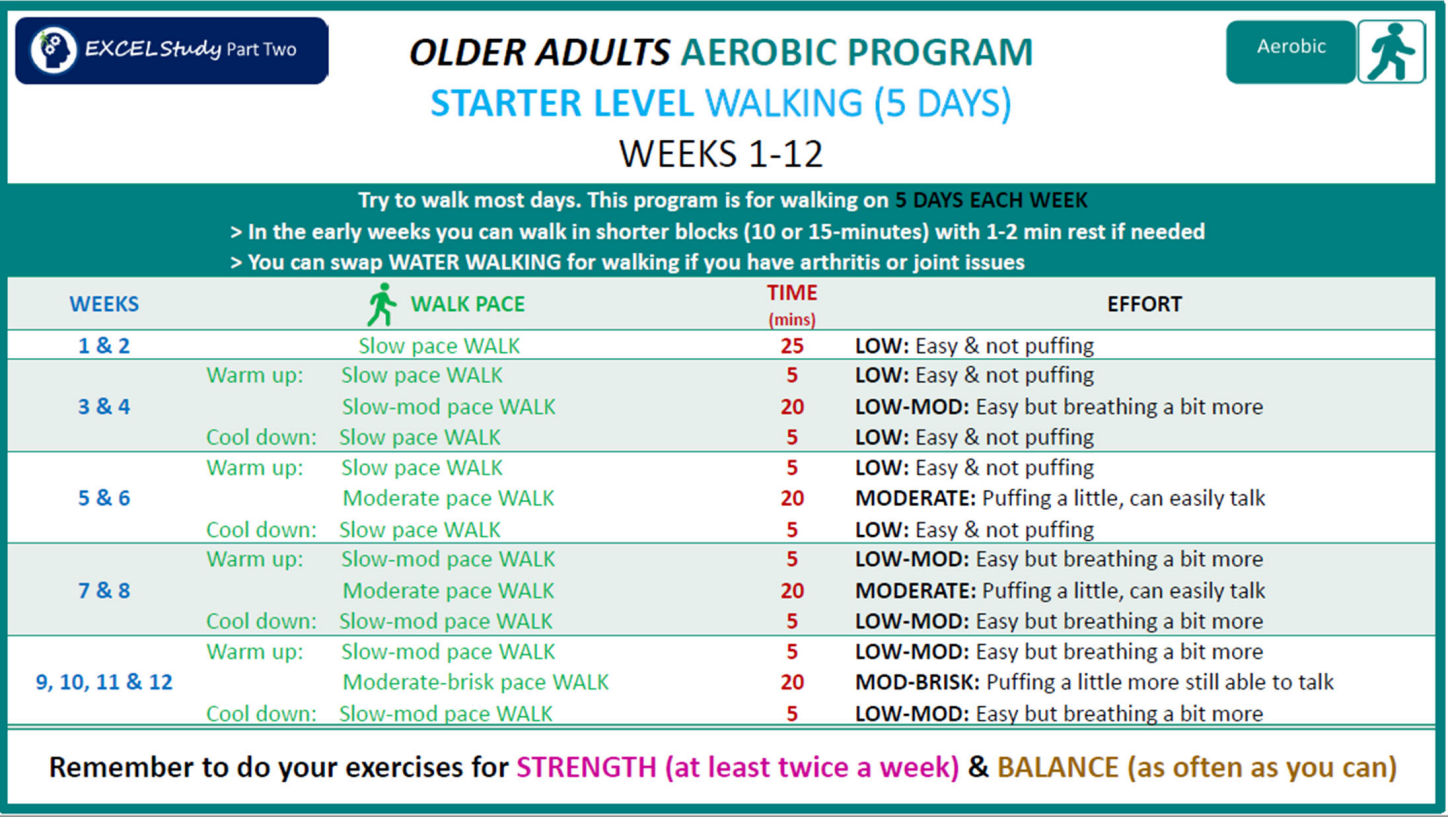
Example of an aerobic program used in the EXCEL intervention. EXCEL: Exercise for Cognitive Health; mod: moderate; MOD: moderate.

**Figure 2.**
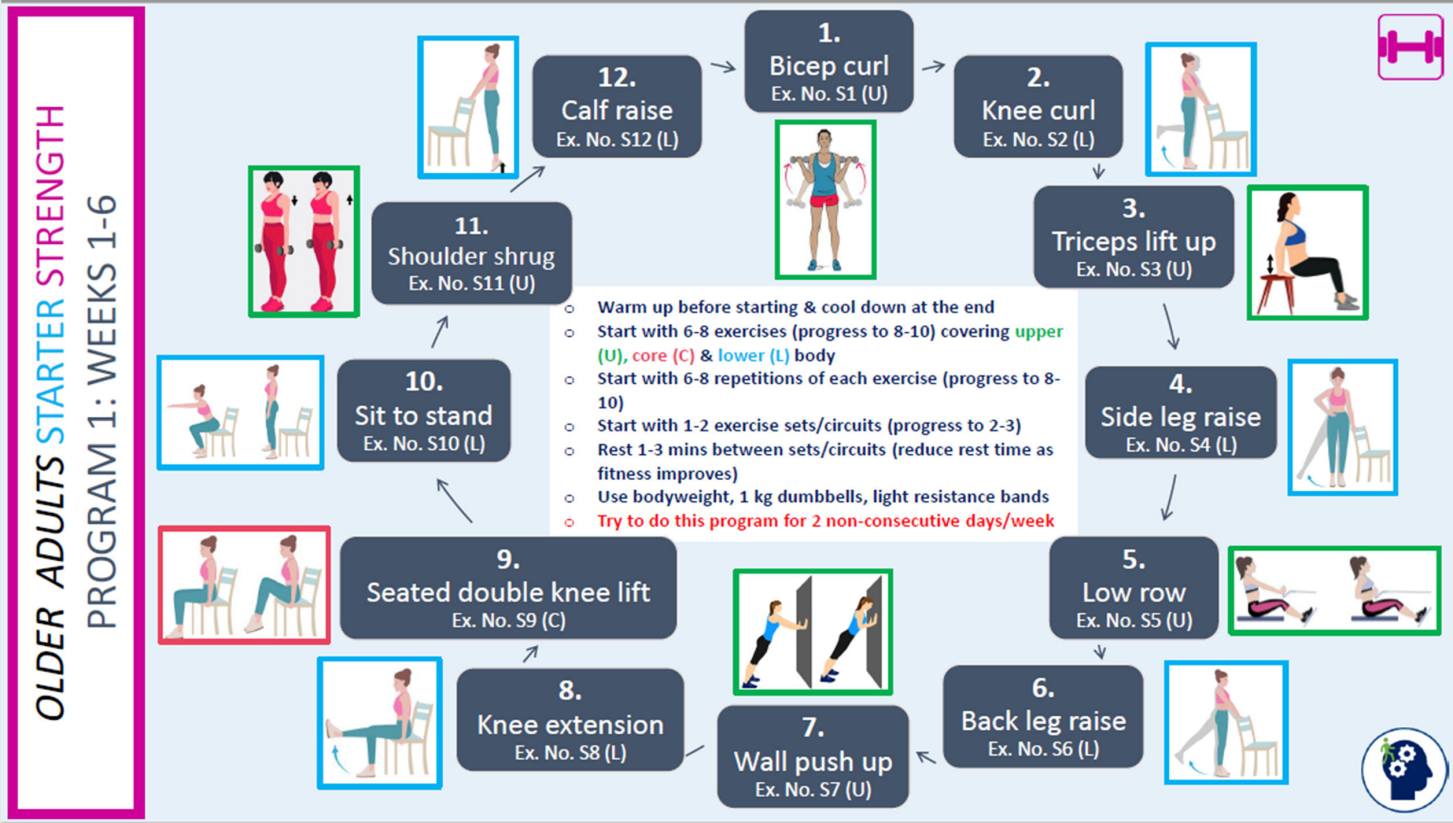
Example of part of a strength program used in the EXCEL intervention. C: core; Ex. no.: exercise number; EXCEL: Exercise for Cognitive Health; L: lower; U: upper.

**Figure 3.**
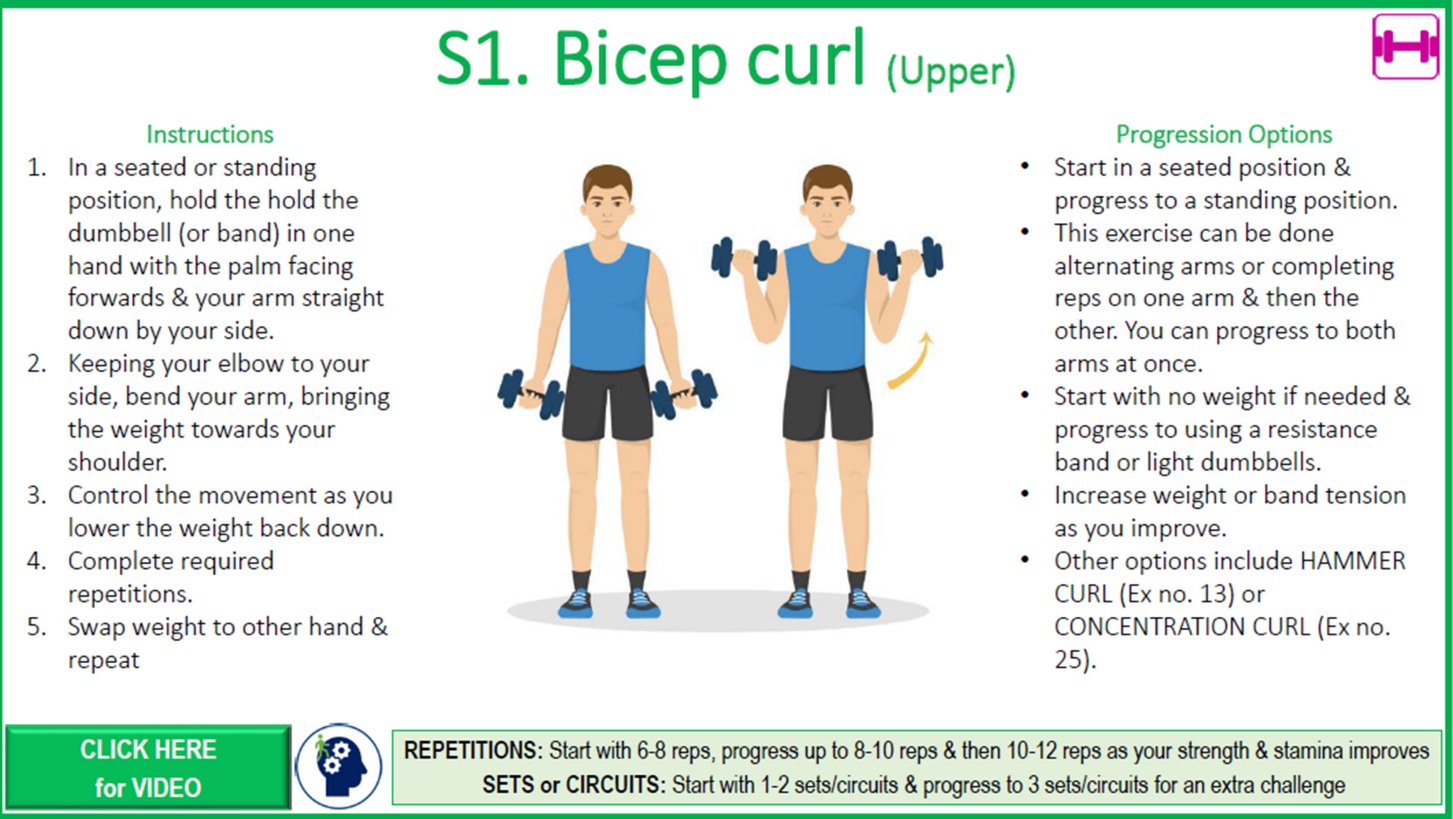
Example of a detailed exercise page in an EXCEL study strength program. Ex. no.: exercise number; EXCEL: Exercise for Cognitive Health; reps: repetitions.

We developed a PA guide incorporating aerobic, strength, and balance programs tailored to 2 age streams: 45‐59 years (based on the standard national PA guidelines [14]) and 60‐80 years (based on national guidelines for older adults with MCI or subjective cognitive decline [12]). While warm-up and cool-down programs were identical for both streams, aerobic and strength programs were customized. Within each stream, participants received either a starter or intermediate program based on their health, abilities, and current activity level. Strength programs differed slightly between males and females. All older age stream participants received a balance program with options for progression. Core program content was developed from successful past studies, including the INDIGO (Individual Goal-Setting and Volunteer Mentors) study [38] and work by Yang et al [39]. The PA guide (Figure 1) provided structured guidance on increasing duration, intensity, number of sessions, and resistance or weights to meet the PA guidelines in 12 weeks. The program incorporated flexibility, with progression guides suggesting rather than prescribing exercises. Each warm-up and cool-down, strength, and balance program included summary pages for easy reference (Figure 2), plus exercise-specific pages detailing performance instructions, pictures, and links to YouTube (Google LLC) demonstration videos (Figure 3). Along with the PA guide and program, participants received a wearable wrist activity tracker, dumbbells, and resistance bands via post if they did not already own this equipment. Participants had access to an online PA diary hosted on the REDCap (Research Electronic Data Capture; Vanderbilt University) platform [40], which could be printed in hard copy or PDF format upon request.

### Coaching or Support

The online support structure consisted of 3 types of sessions as outlined below.

The introduction session was an approximately 70-minute online videoconference session that familiarized participants with key sections of the PA guide, including guidelines and safety information. Participants received personalized directions from an exercise specialist (JS) and were able to ask questions.

Of fortnightly catch-up sessions, there were online sessions (20 min or less) with EXCEL coaches, who checked participants’ diaries, discussed difficulties, and used behavior change strategies tailored to individual needs [22]. These strategies included education, motivation, incentivization, reinforcement, training, environmental restructuring, modeling, and enablement. The 4 EXCEL coaches came from various backgrounds, including psychology, PA, social work, and occupational therapy.

Of exercise specialist sessions, there were as-needed online sessions with the team exercise science specialist (JS) provided extra assistance on exercise elements of the program for participants who requested or required additional support.

### Procedure

**Figure 4.**
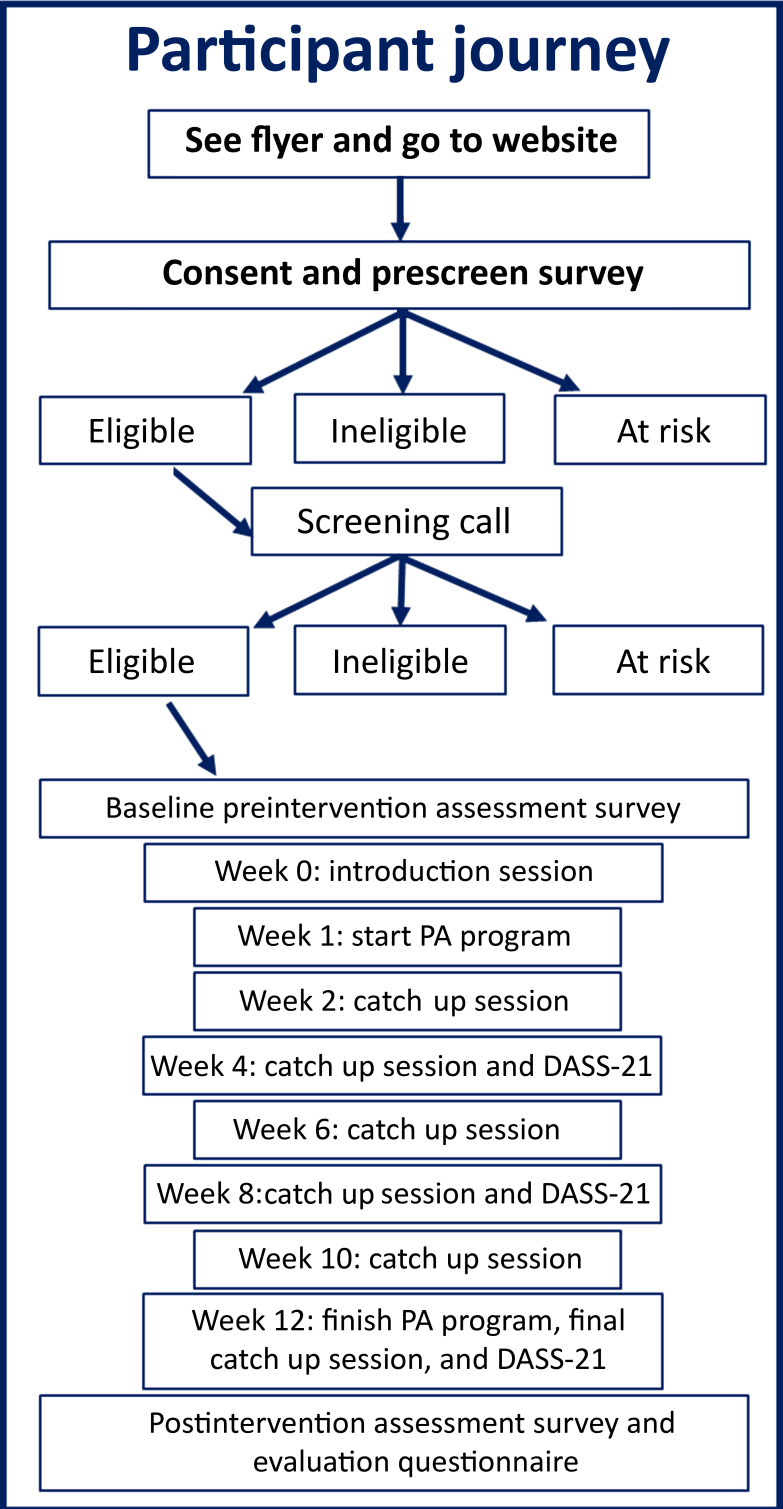
The EXCEL study participant flowchart. DASS-21: Depression Anxiety Stress Scale-21; EXCEL: Exercise for Cognitive Health; PA: physical activity.

Figure 4 summarizes the participant procedure. Participants completed a consent form and prescreening survey and proceeded to a screening call (50 min), completing additional measures to confirm eligibility. Eligible participants were assigned to a PA program and scheduled for an introductory session before completing self-report baseline measures (≈40 min). Their general practitioner was notified of their involvement in the PA intervention. Participants completed the DASS-21 again at weeks 4 and 8 (each ≈5 min). Safety was prioritized, with participants scoring in the severe range or higher on any DASS-21 subscale contacted using a safety protocol script. Fortnightly consultation sessions were held between EXCEL coaches and the research team, which consisted of psychiatrists (NTL, TWHC, TR, and EC), a clinical psychologist (KAE), and exercise physiologists (KLC and JS), to monitor participant safety. Following the 12-week program, participants completed self-report postintervention measures (≈40 min) and an evaluation survey (≈8 min) via Zoom.

### Outcome Measures

Outcome measures are summarized in Table 1. The primary outcomes reflected successful uptake of age-appropriate PA guidelines, specifically meeting the aerobic recommendation of at least 150 minutes per week of moderate or vigorous intensity activity at follow-up and achieving the full set of PA guidelines encompassing aerobic, strength, and balance activities. Secondary outcomes included acceptability, feasibility, and safety, changes in general dementia risk levels, changes in total moderate-to-vigorous PA that incorporates everyday activities, changes in mental health symptoms, and progression through stage of change. Finally, 2 categories of mechanism-focused outcomes were evaluated. The first relates to potential cognitive mechanisms underpinning exercise behavior change, including shifts in knowledge, motivation, and self-efficacy from pre- to postintervention. The second examined potential mechanisms associated with general dementia risk-reduction behavior change over the same period.

**Table 1.** Outcome variables.

Outcome measures	Details
Primary outcomes	Data for primary outcomes were taken from the CHAMPS^a^ questionnaire [41]. For assessing guideline uptake, only purposeful PA^b^ was included, that is, PA done with the purpose of doing exercise. This excluded incidental or everyday PAs, such as gardening, housework, etc.
Uptake of aerobic PA guidelines	Measured by proportion of participants completing 150 minutes or more per week of at least moderate intensity aerobic PA (purposeful PA not including incidental or everyday activities) pre- and postintervention.
Uptake of full PA guidelines	Measured by the proportion of participants completing 150 minutes or more of at least moderate intensity aerobic PA (purposeful PA not including incidental or everyday activities) as well as 2 or more strength training sessions and at least 1 balance session (older adults only) per week pre- and postintervention.
Secondary outcomes	Data for secondary outcomes were taken from a range of tools listed below.
Acceptability, feasibility, and safety	Measured by retention (intervention withdrawals or completions) using a postintervention evaluation questionnaire (developed by the research team to evaluate enjoyment, usefulness, and usability of various elements of the intervention). Safety was assessed by successful monitoring by a clinical panel using a system of email alerts and a safety protocol.
General dementia risk levels	Measured by dementia risk and cognitive health scores pre- and postintervention. This tool is a risk assessment calculator used to understand an individual’s risk of developing dementia (CogDRisk) [42].
Change in PA	Measured by overall minutes of moderate to vigorous intensity PA (including everyday relevant activities) pre- and postintervention using CHAMPS [41]. Measured by overall minutes of any intensity PA (including everyday relevant activities) pre- and postintervention using CHAMPS [41]. Measured by adherence to PA guidelines in 3 domains (aerobic, strength, and balance) over 12 weeks using self-report PA diary data.
Change in mental health symptoms	Measured by depression, anxiety, and stress scores pre- and postintervention. This instrument rates these 3 dimensions of mental health (DASS-21^c^) [29].
Changes in change readiness	Measured by stages of change scores pre- and postintervention including any shift in change stage. This tool assesses an individual’s readiness to change a specific behavior (PA) by evaluating their agreement with statements corresponding to different stages of change: precontemplation, contemplation, preparation, action, and maintenance (modified SOC^d^ [43]).
Change in potential exercise-related cognitive mechanisms of exercise behavior change	Measured by self-efficacy for exercise scores pre- and postintervention. This 9-item scale measures an individual’s confidence in their ability to exercise 3 times per week for 20 minutes in the context of 9 different situations that may interfere with exercise. Items are rated from 0 to 10; 0 represents not confident and 10 represents very confident (modified SEES^e^ [44]). Measured by outcome expectations for exercise scores pre- and postintervention. This 13-item scale includes positive and negative subscales and measures the strength of outcome expectations for exercise. It uses a Likert scale, ranging from 1 (strongly disagree) to 5 (strongly agree; modified OEES-2^f^ [45]). Measured by exercise benefits and barriers for scores pre- and postintervention. This 43-item scale measures perceptions about benefits or barriers to exercising and uses a Likert-type response ranging from 4 (strongly agree) to 1 (strongly disagree; modified EBBS^g^ [46]). Measured by action planning for exercise scores pre- and postintervention. This scale is used to measure the extent to which individuals plan and commit to actions related to their goals and uses a 6-point Likert scale. Participants respond to items that question specific aspects of planning (ie, “I have a clear plan for how to achieve my goal”; APS^h^ [47]).
Change in potential cognitive mechanisms of DRR^i^ related behavior change	Measured by dementia knowledge scores pre- and postintervention (DKAS-subscale D^j^ [48]). Measured by motivation to change dementia risk behavior scores pre- and postintervention. This 27-item scale assesses beliefs and attitudes toward DRR and uses a 5-point Likert scale ranging from 1 (strongly disagree) to 5 (strongly agree; MCLHB-DRR^k^ [49]).

a CHAMPS: Community Health Activities Model Program for Seniors.

b PA: physical activity.

c DASS-21: Depression Anxiety Stress Scale short form.

d SOC: Stages of Change questionnaire.

e SEES: Self-Efficacy for Exercise Scale.

f OEES-2: Outcome Expectations for Exercise Scale.

g EBBS: Exercise Benefits and Barriers Scale.

h APS: Action Planning Scale.

i DRR: dementia risk reduction.

j DKAS-subscale D: Dementia Knowledge Assessment Scale.

k MCLHB-DRR: Motivation to Change Lifestyle and Health Behaviors for Dementia Risk Reduction Scale.

### Statistical Analysis

All quantitative data analyses were conducted using SPSS (version 29; IBM Corp) and R (version 4.1; R Foundation). The *lme4* package was used for the mixed-effects modeling [50], and the *emmeans* package was used to calculate the effect sizes [51], while the *coin* package was used for the asymptotic marginal homogeneity test [52]. Standard assumption checks were undertaken for the linear mixed-effects analyses covering residuals, random effects, variance homogeneity, multicollinearity, influence, and missing data patterns and found no departures that would likely affect the findings. Given the single-group pre-post design, key assumptions required for valid causal mediation analysis, including the ability to rule out unmeasured confounders and separate intervention effects from natural temporal change, could not be met. Therefore, we examined pre-post changes in hypothesized mediators descriptively rather than conducting formal mediation analysis. Descriptive statistics (means, SDs, frequencies, and proportions) were used to describe demographic and outcome measures. The Marginal Homogeneity test, an extension of the McNemar test, was used to compare the proportion of participants who met PA guidelines at baseline to those who met the PA guidelines at postintervention, with the sample restricted to those participants with both baseline and follow-up data. For continuous variables, linear mixed-effects models were used to compare changes in outcome scores from baseline to follow-up, accounting for missing data at follow-up. The models included a fixed effect of time and a random intercept for participants. The Kenward-Roger degrees of freedom approximation was used to calculate *P* values, CIs, and effect sizes. For these models, Cohen *d* effect size was calculated as the mean difference between baseline and follow-up scores divided by the square root of the sum of the intercept (participant) and residual random effect variances [53]. Cohen *d* effect sizes were interpreted as small (*d*=0.2), medium (*d*=0.5), and large (*d*≥0.8). The Benjamini and Yekutieli [54] approach was used to correct for multiple testing of the primary outcomes and secondary outcomes, respectively. For these analyses, an α of .05 was used for statistical inference.

### Adherence to PA Guidelines

Participants’ self-reported diaries collected over the 12-week intervention period were used to assess adherence to PA guidelines. No consensus exists on defining PA adherence; similar studies commonly use meeting 2/3 or more of the prescribed PA [55]. We will apply two thresholds: (1) full adherence, defined as meeting the complete guideline each week (>150 min/wk moderate-plus aerobic PA, >2 strength sessions/wk, and for older adults >1 balance session/wk); and (2) 2/3 adherence, defined as >100 minutes per week of aerobic PA, ≥1 strength session per week, and ≥1 balance session per week for older adults. Aggregate total adherence over the 12-week intervention period will use a realistic expectation of 8 or more out of a possible 12 weeks’ meeting in full or with 2/3 guidelines.

## Results

### Participant Demographics

**Figure 5.**
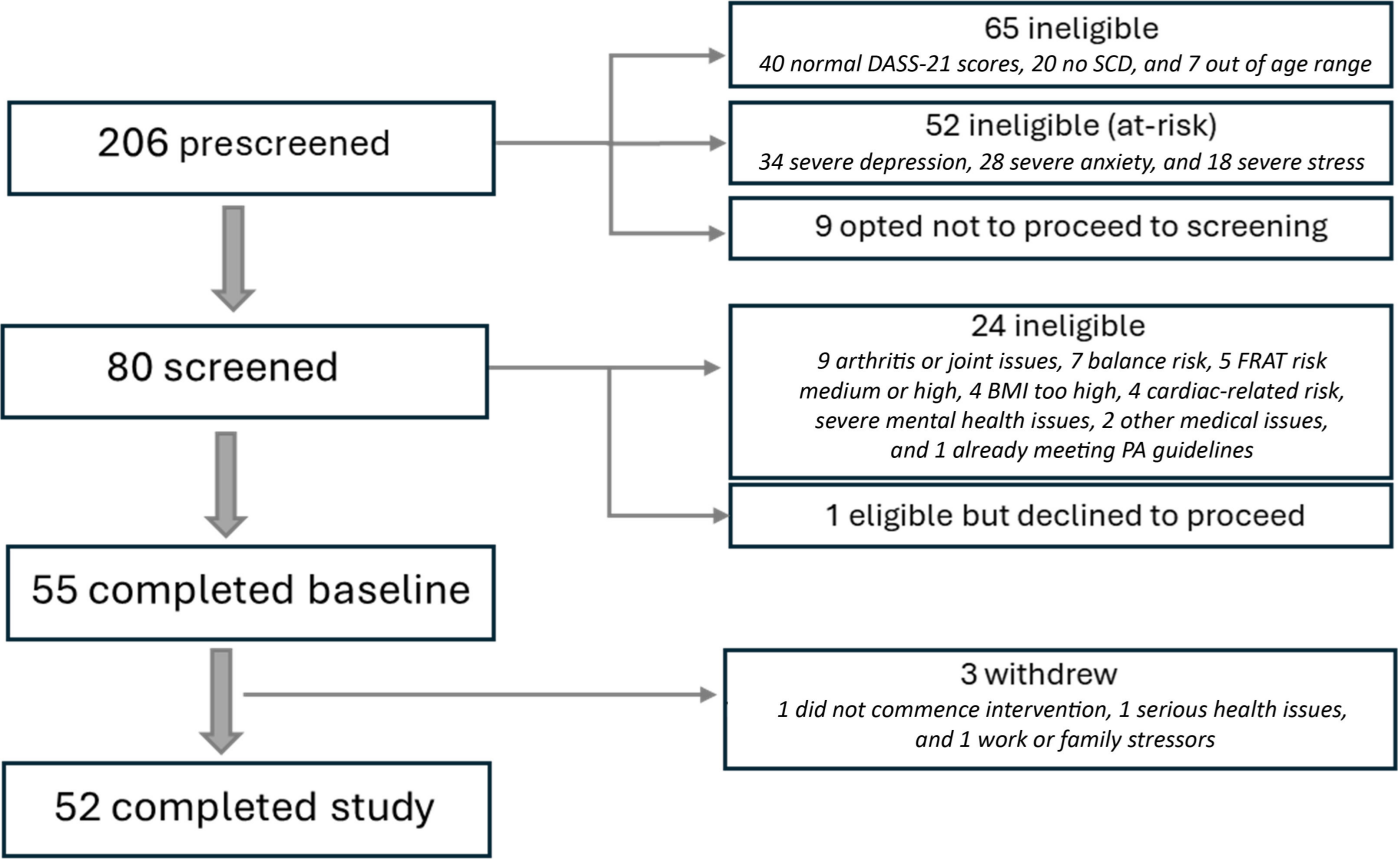
Study recruitment and screening flow diagram. DASS-21: Depression Anxiety Stress Scale short form; FRAT: falls risk assessment tool; PA: physical activity; SCD: subjective cognitive decline.

Figure 5 summarizes this study recruitment and screening process; 55 participants were enrolled and completed baseline assessment (46 female/9 male; mean age at screening 62.2, SD 7.6 y). Demographic characteristics are presented in Table 2.

**Table 2.** Participant demographic characteristics at baseline.

	Baseline (n=55)
Gender, n (%)	
Man	9 (16)
Woman	46 (84)
Age (y)	
Mean (SD)	62.2 (7.6)
Older age group (60‐80), n (%)	43 (78)
Middle-aged group (45‐59), n (%)	12 (22)
Country of birth, n (%)	
Australia	33 (60)
Other English-speaking (eg, New Zealand, the United Kingdom, and the United States)	10 (18)
Other	12 (22)
Speaks a language other than English at home, n (%)	
No	52 (95)
Yes	3 (5)
Education, n (%)	
Bachelor’s degree or higher	43 (78)
Work status, n (%)	
Retired	16 (29)

### Preliminary Analysis

We performed sensitivity analyses adding age, gender, baseline depression score, and baseline total minutes of PA as covariates; their inclusion had a negligible effect on the estimated mean difference and effect size and did not meaningfully change the CIs, indicating the main results are robust to these potential confounders, and the full results are reported in Multimedia Appendix 1.

### Primary Outcomes

#### Uptake of Aerobic PA Guidelines

We used the more rigorous measure of aerobic PA (ie, purposeful aerobic PA which did not include everyday activities, eg, housework, gardening, etc) to gauge the uptake of aerobic PA guidelines via the provided aerobic PA programs. For changes in broader measures of aerobic PA, see Table 3. At baseline, 44% (24/55) of participants met the purposeful aerobic PA guidelines (150 min of moderate or 90 min of vigorous) aerobic PA per week. At postintervention, this figure increased by 11% to 55% (30/55) participants meeting purposeful aerobic PA guidelines (Table 4). Of those participants who did not meet full purposeful aerobic guidelines at baseline, at postintervention, 37% (11/52) of participants met these guidelines (*P=*.06; Table 5).

**Table 3.** Baseline and follow-up for secondary outcome measures. There were 55 participants at baseline and 52 participants at postintervention. *P* values were adjusted for multiple testing.

Secondary outcomes	Baseline, mean (SD)	Postintervention, mean (SD)	Mean differences (SE; 95% CI)	*P* value	Cohen *d* (95% CI)
CogDRisk total risk score	4.80 (4.88)	3.10 (4.60)	−1.52 (0.43; −2.39 to −0.65)	.008	−0.32 (−0.50 to −0.13)
Overall min mod/ vigorous PA^a^	380.45 (347.27)	495.29 (370.14)	114.40 (37.39; 39.37 to 189.44)	.02	0.32 (0.10 to 0.54)
Overall minutes of all PA^b^	32.55 (449.07)	936.06 (588.71)	205.25 (54.93; 95.02 to 315.48)	.005	0.39 (0.17 to 0.62)
DASS-depression^c^	11.02 (4.68)	4.92 (4.61)	−6.07 (0.68; −7.44 to −4.70)	<.001	−1.31 (−1.70 to −0.92)
DASS-anxiety	5.35 (3.98)	2.15 (3.01)	−3.15 (0.54; −4.22 to −2.07)	<.001	−0.89 (−1.23 to −0.54)
DASS-stress	14.18 (4.86)	7.69 (6.15)	−6.51 (0.84; −8.20 to −4.82)	<.001	−1.18 (−1.56 to −0.80)
Stages of change	3.73 (1.18)	4.27 (0.82)	0.54 (0.18; 0.19 to 0.89)	.02	0.53 (0.17 to 0.88)
SEES^d^ total self-efficacy score	56.78 (17.71)	55.08 (18.03)	−1.88 (2.22; −6.33 to 2.56)	≥.99	−0.10 (−0.35 to 0.14)
OEES^e^-POESS^f^	4.20 (0.60)	4.40 (0.64)	0.20 (0.06; 0.33 to 0.07)	.02	0.33 (0.54 to 0.11)
OEES-NOESS^g^	4.34 (0.69)	4.49 (0.60)	−0.12 (0.08; −0.28 to 0.03)	.47	−0.19 (−0.42 to 0.05)
EBBS benefit^h^ average score	3.19 (0.40)	3.23 (0.48)	0.04 (0.05; −0.06 to 0.14)	≥.99	0.09 (−0.13 to 0.30)
EBBS barrier^i^ average score	1.94 (0.39)	1.85 (0.43)	−0.09 (0.05; −0.18 to 0.00)	.30	−0.22 (−0.45 to 0.01)
Action Planning Scale average score	2.88 (1.58)	3.96 (1.64)	1.07 (0.24; 0.58 to 1.56)	.001	0.66 (0.34 to 0.99)
DKAS^j^ average subscale D score	0.98 (0.45)	1.07 (0.48)	0.09 (0.05; −0.02 to 0.20)	.47	0.19 (−0.04 to 0.43)
MCLHB^k^ average total score	3.40 (0.39)	3.36 (0.34)	−0.05 (0.04; −0.13 to 0.03)	.92	−0.13 (−0.34 to 0.09)

a Min mod/ vigorous PA: minimum moderate-vigorous physical activity.

b PA: physical activity.

c DASS: Depression Anxiety Stress Scale short form.

d SEES: Self-Efficacy for Exercise.

e OEES: Modified Outcome Expectancy for Exercise Scale.

f POESS: Positive Outcome Expectancy for Exercise.

g NOESS: Negative Outcome Expectancy for Exercise.

h EBBS benefit: Exercise Benefits/Barriers Scale – Benefits subscale.

i EBBS barrier: Exercise Benefits/Barriers Scale – Barrier subscale.

j DKAS: Dementia Knowledge Assessment Scale.

k MCLHB: Motivation to Change Lifestyle and Health Behaviors for Dementia Risk Reduction.

**Table 4.** Proportion of cohort meeting aerobic and purposeful guidelines at baseline and 12 weeks.

	Baseline (n=55)	12-weeks (n=55)
Meeting full purposeful aerobic guidelines, n (%)		
No	31 (56)	22 (40)
Yes	24 (44)	30 (55)
Withdrew	0 (0)	3 (6)
Meeting full purposeful total guidelines, n (%)		
No	52 (94)	28 (51)
Yes	3 (6)	24 (44)
Withdrew	0 (0)	3 (6)

**Table 5.** Physical activity guidelines transitions from baseline to postintervention (n=52).

Baseline	Postintervention		
	No	Yes	Total
Meeting full purposeful aerobic guidelines, n (%)			
No	19 (64)	11 (37)	30 (100)
Yes	3 (14)	19 (86)	22 (100)
Meeting full purposeful total guidelines, n (%)			
No	28 (56)	22 (44)	50 (100)
Yes	0 (0)	2 (100)	2 (100)

#### Uptake of Full PA Guidelines

Meeting the full PA guidelines includes aerobic PA as described above, as well as 2 or more strength sessions per week, and for older adults, balance exercises as often as possible (at least once per week). At baseline, 52 of 55 (94%) participants were not meeting the full purposeful PA guidelines, and postintervention, this figure had dropped to just over half (28/55) of participants who were not meeting these guidelines (Table 4). Likewise, of those participants who did not meet full purposeful total guidelines at baseline, 44% (22/52) of participants met these guidelines at postintervention (*P*<.001; Table 5).

### Secondary Outcomes

#### Acceptability, Feasibility, and Safety

Postintervention feedback was received from 94% (49/52) of intervention completers. Further, 51 of 52 (98%) participants found the program to be useful, and all 52 participants stated they would recommend such a program to a friend, demonstrating acceptability. At week 12, a total of 96% (50/52) of participants expressed an intention to continue with PA beyond the EXCEL program. Fortnightly catch-up sessions were deemed the most helpful component; all 52 participants rated this support as helpful to very helpful, with 79% (41/52) of participants rating it as very helpful. Catch-up call attendance was very high; 89% (46/52) of participants attended all 6 calls. The remaining 6 participants missed only 1 catch-up session. Most liked that the program was provided online and was completed at home (50/52, 96% of participants, and 48/52, 92% of participants, respectively). Retention rates were notably high; 52 of 55 (95%) participants completed this study, providing support for program feasibility. Zoom technology was used for 91% (189/208) of all catch-up sessions, with telephone a practical back-up option for the remaining 9% (19/208) of sessions. Technology issues were uncommon; 76% (40/52) of participants reported seldom or never encountering problems. The intervention was supported by a comprehensive monitoring system that included 44 email alerts from coaches to the medical team, 35 queries or requests from coaches to the PA team, 3 individual participant sessions via Zoom with an exercise specialist, and several case conferences, ensuring participant safety and supporting adherence.

#### Dementia Risk Levels

The intervention was associated with a reduction in dementia risk, with an average decrease of 1.52 (SE 0.43) points on the CogDRisk (95% CI 0.65 to 2.39, *P*=.008; see Table 3). This is consistent with a small to moderate effect (*d*=0.32, 95% CI 0.13 to 0.50).

#### Moderate to Vigorous Intensity PA and All PA

Moderate to vigorous intensity PA levels, measured by the CHAMPS, increased by an average of 114.40 (SE 37.39; 95% CI 39.37 to 189.44) minutes per week from baseline to postintervention. The effect size was small to moderate (*d*=0.32; Table 3). This included any PA performed at moderate or higher intensity, encompassing all types (eg, aerobic or strength-based PA) and any purpose, whether purposeful (undertaken with the intention of being active) or incidental or everyday activity (eg, gardening). Likewise, all PAs increased by an average of 202.25 (SE 54.93; 95% CI 95.02 to 315.48) minutes from baseline to postintervention; the effect size was small to moderate (*d*=0.39; Table 3). This included any intensity (low to very high), any type (aerobic, strength, and balance), and any purpose (purposeful and incidental).

#### PA Guideline Adherence

Weekly adherence data is summarized in Table 6. All component guidelines adherence was highest during weeks 3 to 6 (>70%; at least 36 of the 52 participants), and all component guidelines were met by 31% (16/52) of participants for 8 or more weeks. Average weekly volume of moderate intensity aerobic PA fluctuated over the 12 weeks but did not drop below the recommended 150 minutes per week. Weekly strength session numbers were stable across the intervention and consistently exceeded the minimum of 2 sessions per week, with an average of 2.2 (SD) sessions per week. Weekly adherence to the balance guidelines was only applicable for older adults and was high throughout the intervention. Weekly numbers of balance PA sessions were relatively stable over the 12 weeks, with an average of 4.1 (SD) sessions per week, ranging from 3.8 to 4.3 sessions per week.

**Table 6.** Weekly adherence to PA^a^ guideline components over the 12-week intervention.

PA component and weekly adherence	Full guideline	≥2/3 guideline
All components^b^		
Highest, n (%)	>36 (>70) – Weeks 3‐6	41 (79) – Week 4
Lowest, n (%)	20 (39) – Week 12	28 (54) – Week 8
Mean (SD)	23 (44)	34 (65)
Aerobic PA^b^		
Highest, n (%)	33 (64) – Week 3	46 (89) – Week 4
Lowest, n (%)	23 (44) – Week 9	32 (62) – Week 9
Mean (SD)	28 (54)	37 (71)
Strength PA^b^		
Highest, n (%)	≈42 (≈80) – Weeks 1, 3‐5	≥47 (>90) – Weeks 1, 3‐5
Lowest, n (%)	33 (64) – Week 12	41 (79) – Week 12
Mean (SD)	38 (74)	45 (87)
Balance PA^c^		
Highest, n (%)	43 (100) – Weeks 7, 10	—^d^
Mean (SD)	40 (93)	—

a PA: physical activity.

b n=52.

c n=43 (balance component for older adults only).

d Not available.

#### Mental Health Symptoms

Analysis of mental health outcomes measured using the DASS-21 showed improvements across all dimensions. Effect sizes for changes in depression, anxiety, and stress scores were large at 1.31, 0.89, and 1.18, respectively (Table 3). Depression decreased by 6.1 points (SE 0.7; 95% CI 4.7 to 7.4; *P*<.001), anxiety decreased by 3.2 points (SE 0.5; 95% CI 2.1 to 4.2; *P*<.001), and stress decreased by 6.5 points (SE 0.8; 95% CI 4.8 to 8.2; *P*<.001). DASS-21 subscale scores can be multiplied by 2 to allow comparison with established DASS-42 severity norms. Using this interpretive framework, mean depression and stress scores shifted from the severe to mild range, and anxiety scores from the moderate to normal range.

#### Exercise-Related Cognitive Mechanisms (Change Readiness, Self-Efficacy, Outcome Expectations, Benefits and Barriers, and Planning)

There was strong evidence of an intervention effect on change readiness (*χ*^2^_4_=16.1, *P*<.003). As shown in Table 7, a total of 27 of 52 (52%) participants transitioned from a lower stage at baseline of change to a higher stage of change at postintervention, and 29 of 52 (56%) participants remained in the same stage of change at postintervention. For the latter group, however, there was a ceiling effect whereby 15 of the 29 participants who started in the highest stage of change at baseline could not transition to a higher stage of change at postintervention. Notably, all participants who started in stage 2 or stage 3 at baseline progressed to a later stage of change postintervention. There were 8 of 52 (15%) of participants who transitioned from a higher stage of change at baseline to a lower stage of change post intervention.

**Table 7.** Stages of change transition from baseline to postintervention (n=52).

Baseline	Postintervention					
	1	2	3	4	5	Total
Do not intend, n (%)	0 (0)	0 (0)	0 (0)	1 (100)	0 (0)	1 (100)
Intend to start, n (%)	0 (0)	0 (0)	1 (14)	6 (86)	0 (0)	8 (100)
Started but not regular, n (%)	0 (0)	0 (0)	0 (0)	13 (77)	4 (24)	17 (100)
Have started regular, n (%)	0 (0)	0 (0)	2 (33)	1 (17)	3 (50)	6 (100)
Currently exercise regularly, n (%)	1 (5)	1 (5)	0 (0)	4 (19)	15 (71)	21 (100)

There were no significant changes in self-efficacy or perceived barriers and benefits to exercise (Table 3). However*,* our data demonstrated an improvement in both planning and positive outcome expectation postintervention. There was a moderate effect of the intervention for average planning (*d*=0.66, 95% CI 0.34 to 0.99), with an average increase of 1.07 points (SE 0.24; 95% CI 0.58 to 1.56, *P*=.001) from baseline to postintervention. Furthermore, there was a small to moderate intervention effect on positive outcome expectations for exercise (*d*=0.33, 95% CI 0.11 to 0.54), with an average increase of 0.20 points (SE 0.06; 95% CI 0.07 to 0.33, *P*=.02).

#### DRR Cognitive Mechanisms (Dementia Knowledge and Motivation)

There was no change in dementia knowledge (mean difference=0.09, SE 0.5; 95% CI −0.20 to 0.02, *P=*.47) and attitudes and beliefs toward lifestyle adaptations for DRR (mean difference=0.05, SD, 95% CI –0.03 to 0.13, *P*=.92) from baseline to postintervention (Table 3).

## Discussion

### Principal Findings

These findings support our hypothesis that the EXCEL program, a 12-week digitally delivered, tailored PA intervention, could effectively facilitate middle-aged and older Australians experiencing mild to moderate mental health symptoms alongside cognitive concerns to meet PA guidelines. The program was acceptable, feasible, and safe and was associated with reductions in both dementia risk and mental health symptoms and progression through stages of change. Changes to action planning and positive outcome expectancies were observed and present potential mechanisms of action, reflecting components of the behavior change model that informed the intervention’s design [14]. Given the well-documented challenges in engaging this population in sustained PA and the critical importance of PA for DRR, these results are particularly encouraging.

### Comparison With Prior Work

EXCEL was highly efficacious; nearly half of the cohort (24/55, 44% participants) met PA guidelines at the end of the program, up from 6% (3/55) of participants at baseline. The increases in PA are particularly encouraging given the low proportion meeting PA guidelines at baseline (<6%), compared to the Australian population levels of 22.4% of adults and 33.4% of older adults [56]. Low baseline PA levels in our cohort fit with evidence that people with mental health disorders are significantly less likely to meet PA guidelines compared to controls [57]. This further underlines the critical importance of interventions that are tailored to support this cohort at high risk for dementia. Retention rates (95%) and positive feedback from participants (ie, 98% found it useful and 100% would recommend it to others) demonstrate that the program was well-received. High levels of acceptability might be attributed to the individually tailored nature of the intervention, which was designed based on comprehensive formative research incorporating the preferences and needs of the target demographic [15]. It is also relevant to note that EXCEL was conducted in part during the COVID-19 lockdowns, which may have increased participants’ involvement due to a reduction in alternative activities and distractions. Evidence suggests that while COVID-19 had a mixed impact on research participation, research that used PA was associated with increased involvement [58].

Safety was a central focus of our design, particularly given the online delivery. While the safety, scalability, and effectiveness of digital PA interventions have been well documented in younger adults (see umbrella review by Russell et al [59]), there remains a gap in the literature regarding older adults. We used a robust system of support and monitoring that included email alerts and professional consultations that managed physical and mental health risk successfully. Our current results, taken together with data that show the majority of middle-aged and older adults are online (98% of Australians aged 65‐74 y and 94% of those aged 75 y + [60]), suggest that age should not be seen as a barrier to accessing safe and effective online PA interventions. Another advantage suggesting future scalability was that coaches were not required to have mental health credentials or exercise physiology expertise themselves to be highly effective. The safety of our trial and the training and support of our coaches did, however, require qualified mental health and exercise physiology clinicians in an oversight capacity, which has implications for real-world implementation.

A significant reduction in dementia risk was observed over the 12 weeks, as measured by the CogDRisk, a validated online measure that assesses individualized risk factors of dementia in various population settings [42,61]. These results support a developing literature showing that PA reduces dementia risk in other high-risk groups, including those with new-onset type II diabetes [62]. Baseline dementia knowledge in our cohort was low and was not modified by our intervention, which is to be expected given that this was not an aim of the current study. This does demonstrate, however, that it is not essential to increase dementia knowledge to reduce dementia risk, which may be particularly pertinent for individuals with cognitive difficulties. Nonetheless, integrating additional psychological or social support structure components could further enhance the intervention’s impact on self-efficacy and sustained behavior change.

We evaluated whether the intervention impacted mental health symptoms and found a significant and clinically meaningful improvement across all DASS-21 dimensions. After 12 weeks, our cohort’s anxiety levels had returned to within the normal range, while depression and stress symptoms were now mild. This is a meaningful change from the extremely severe (stress), severe (depression), and moderate (anxiety) average symptom scores observed 12 weeks earlier, at baseline. Our findings add to the established literature showing the important positive effect that exercise can play in improving mood; a finding reflected in clinical practice guidelines in the United States, United Kingdom, and Australia [63-65]. A recent meta-analysis by Noetel et al [66] showed exercise to be equally effective for people with and without comorbidities, and with different baseline levels of depression. Their findings reflected over 200 studies [66] and suggest that walking and strength training are among the most effective exercises for depression reduction, in line with PA guidelines that underlie EXCEL. While evidence for the anxiety-reducing effect of PA is currently less consistent (see Chong et al [67] for a meta-analysis), we were encouraged to see that PA reduced anxiety levels in our cohort.

### Potential Mechanisms of Action

EXCEL was designed to specifically target proposed mechanisms of action for optimizing engagement in this cohort, including emotional regulation, self-efficacy in existing skills, and the capacities to act on intentions despite barriers, and we measured these potential cognitive mechanisms pre- and postintervention. Our findings showed a small to moderate, but significant, increase in positive outcome expectations for exercise. Positive outcome expectancy refers to an individual’s belief that PA will lead to certain (positive) outcomes and is 1 of 2 key constructs in social cognitive theory, alongside self-efficacy. Our findings are consistent with previous research showing that (1) positive, rather than negative, outcome expectancy is associated with PA levels, and (2) there is an apparent age-related effect whereby outcome expectancy is more predictive of PA in older adults [68]. Positive outcome expectancies have previously been observed to facilitate the formation of PA intentions [68] and are associated with progression through the stages of change, as seen in this study. Considered within the COM-B framework, positive outcome expectancies may enhance motivation and support the development of strong intentions to engage in PA.

However, transitioning from intention to behavior often relies on effective action planning—that is, setting concrete, detailed plans that outline the when, where, and how of intended PAs [69]. Our findings support this notion. The mental stimulations involved in planning have also been shown to have a self-regulatory effect that may help individuals connect their behavioral responses to future scenarios and account for potential barriers [70]. In this context, our intervention may have supported participants in mentally rehearsing the steps needed to translate PA intentions into action, reducing the intention-behavior gap. This aligns with our findings on the stages of change measure: at baseline, participants primarily scored in the “preparation” stage, characterized by intentions to begin regular exercise or making sporadic attempts to be active. Following the intervention, averages shifted to the “action” stage, reflecting active and consistent engagement in structured exercise. These results suggest that aging adults with cognitive difficulties related to subjective or MCI and co-occurring mental health symptoms may particularly benefit from pragmatic, structured action planning that bridges the gap between intention and behavior.

### Limitations and Future Directions

While our results are promising, they should be interpreted with caution due to the pilot nature of this study, modest sample size, and pre-post design. This study’s design prevented a valid causal mediation analysis, meaning that changes in hypothesized mediators can only be described rather than inferred as causal. Further research using a larger sample and a randomized controlled trial design with mediation analyses is needed to determine whether the proposed mechanisms contribute causally to the observed outcomes. Sensitivity analyses indicated that age, gender, baseline depression score, and overall minutes of PA did not confound the findings. However, technological or digital literacy was not measured, and engagement may have been higher during COVID-19 lockdowns; these unmeasured confounders may limit generalizability to nonpandemic conditions. Despite these limitations, the findings exemplify the utility of tailored interventions and the value of a behavior-change framework. It is recommended that future studies include larger cohorts and longer follow-up periods to replicate findings and evaluate the durability of effects. Given the complexity of factors influencing PA adherence and mental health among aging adults, multicomponent interventions that address both physical and cognitive health holistically are recommended.

### Conclusions

This pilot study demonstrated EXCEL to be effective, acceptable, feasible, and safe. The tailored, PA-delivered PA intervention supported middle-aged and older adults experiencing mild to moderate mental health symptoms alongside cognitive concerns to meet PA guidelines. Important secondary gains were a reduction of dementia risk and clinically meaningful improvements in mental health symptoms, emphasizing the advantages of boosting PA in high-risk groups. Improvements in action planning and outcome expectancy may have contributed to bridging the PA intention-behavior gap, in line with the COM-B model underlying the intervention design. While findings require confirmation in larger controlled trials, the results suggest that EXCEL offers a scalable, evidence-informed model for supporting dementia-risk reduction in adults who face well-documented barriers to engaging in sustained PA.

## Supplementary material

10.2196/80040 Multimedia Appendix 1 Results of sensitivity analysis examining age, gender, baseline physical activity, and baseline depression severity as potential confounders of primary and secondary outcome measures.

## Abbreviations

COM-B: Capability, Opportunity, and Motivation Behavior
DASS-21: Depression Anxiety Stress Scale short form
DRR: dementia risk reduction
EXCEL: Exercise for Cognitive Health
INDIGO: Individual Goal-Setting and Volunteer Mentors
MCI: mild cognitive impairment
PA: physical activity
REDCap: Research Electronic Data Capture
