# Supplementary material for: Tailored Online Physical Activity Coaching for Middle-Aged and Older Adults With Cognitive and Mental Health Concerns: Single-Arm Pre-Post Intervention Study
Source: JMIR Aging. 2026 Mar 6;9:e80040. doi: 10.2196/80040 (PMC12977004; doi:10.2196/80040)
Supplement: Multimedia Appendix 1 — Results of sensitivity analysis examining age, gender, baseline physical activity, and baseline depression severity as potential confounders of primary and secondary outcome measures. [file aging-v9-e80040-s001.pdf]

Table S1. Results of sensitivity analysis examining age, gender, baseline physical activity, and baseline depression severity as potential confounders of primary and secondary outcome measures.

Outcomes	Age		Gender		Physical Activity (Minutes of all PA)		Depression	
	Mdiff (95% CI)	Cohen's d (95% CI)	Mdiff (95% CI)	Cohen's d (95% CI)	Mdiff (95% CI)	Cohen's d (95% CI)	Mdiff (95% CI)	Cohen's d (95% CI)
Primary								
Overall min mod to vigorous intensity PA	115.85 (40.73, 190.97)	0.33 (0.11, 0.55)	113.71 (38.69, 188.73)	0.33 (0.10, 0.55)	-	-	114.55 (39.50, 189.60)	0.32 (0.10, 0.54)
Overall minutes of all PA	208.93 (98.41, 319.46)	0.42 (0.18, 0.66)	204.14 (93.90, 314.37)	0.40 (0.17, 0.63)	-	-	205.40 (95.15, 315.64)	0.39 (0.17, 0.62)
Secondary								
DASS-Depression	-6.04 (-7.41, -4.67)	-1.31 (-1.70, -0.92)	-6.08 (-7.45, -4.71)	-1.32 (-1.71, -0.93)	-5.93 (-7.35, -4.52)	-1.27 (-1.66, -0.89)	-	-
DASS-Anxiety	-3.15 (-4.22, -2.07)	-0.88 (-1.23, -0.54)	-3.14 (-4.22, -2.07)	-0.88 (-1.23, -0.54)	-3.03 (-4.16, -1.90)	-0.86 (-1.21, -0.50)	-3.15 (-4.23, -2.07)	-0.89 (-1.23, -0.54)
DASS-Stress	-6.52 (-8.21, -4.83)	-1.17 (-1.55, -0.80)	-6.50 (-8.19, -4.81)	-1.18 (-1.56, -0.80)	-6.33 (-8.08, -4.59)	-1.14 (-1.52, -0.77)	-6.50 (-8.19, -4.82)	-1.17 (-1.55, -0.80)
Stages of change	0.55 (0.20, 0.91)	0.55 (0.19, 0.92)	0.53 (0.18, 0.89)	0.53 (0.17, 0.88)	0.49 (0.13, 0.86)	0.48 (0.12, 0.85)	0.54 (0.18, 0.89)	0.52 (0.17, 0.88)
APS average score	1.07 (0.58, 1.56)	0.66 (0.33, 0.98)	1.07 (0.58, 1.56)	0.66 (0.34, 0.99)	0.88 (0.39, 1.36)	0.56 (0.24, 0.88)	1.07 (0.58, 1.56)	0.66 (0.33, 0.98)
DKAS average subscale D score	0.09 (-0.02, 0.20)	0.19 (-0.05, 0.43)	0.09 (-0.02, 0.20)	0.19 (-0.04, 0.43)	0.07 (-0.05, 0.18)	0.15 (-0.10, 0.40)	0.09 (-0.02, 0.20)	0.19 (-0.04, 0.43)
MCLHB average total score	-0.05 (-0.13, 0.03)	-0.13 (-0.35, 0.09)	-0.05 (-0.13, 0.03)	-0.13 (-0.34, 0.09)	-0.05 (-0.13, 0.04)	-0.12 (-0.35, 0.11)	-0.05 (-0.13, 0.03)	-0.13 (-0.34, 0.09)
CogDRisk Total risk score	-1.50 (-2.36, -0.64)	-0.32 (-0.52, -0.13)	-1.53 (-2.40, -0.66)	-0.32 (-0.52, -0.13)	-1.47 (-2.41, -0.54)	-0.30 (-0.51, -0.10)	-1.52 (-2.38, -0.65)	-0.32 (-0.50, -0.13)
